# Antibiotic Cement-Coated interlocking Intramedullary Nails in the Treatment of Septic Complex Lower Extremity Reconstruction; A Retrospective Analysis with Two year Minimum Follow up

**DOI:** 10.7150/jbji.46570

**Published:** 2020-05-29

**Authors:** Asim M. Makhdom, Joshua Buksbaum, S. Robert Rozbruch, Rachael Da Cunha, Austin T. Fragomen

**Affiliations:** 1 Foothills Medical Group, Upper Alleghany Health System, Olean, NY and Bradford, PA, USA.; 2 Hospital for Special Surgery, Weill Cornell Medicine, Cornell University, New York, NY, USA.; 3 SUNY Downstate Health Sciences University, College of Medicine, Brooklyn, NY, USA.; 4 Kingston Health Sciences Centre, Queen's University, Kingston, ON, Canada.

**Keywords:** antibiotic cement coated interlocking intramedullary nails, arthroplasty

## Abstract

**Background:** To report on our experience with antibiotic cement coated interlocking intramedullary nails (ACC-IMNs) for limb salvage in septic complex lower extremity reconstruction with a minimum of 2-year follow up.

**Methods:** We retrospectively reviewed the records of all consecutive patients who underwent a limb salvage procedure with ACC-IMNs. We reviewed patients' demographics, the preoperative infecting organism, and host type, time to bone union, complications, limb salvage rates, and infection control rates.

**Results:** There were 28 patients with a mean age of 62 years (range 22-88). The mean follow up period was 40 months (range 28-84). The ACC-IMNs were used in 14 patients (50 %) to achieve knee fusion after failed revisions of infected total knee arthroplasty, in 8 patients (28%) for septic tibial nonunion, and in 6 patients (21%) with ankle fusion nonunions. Bony union/fusion was achieved in 87 % (21/24) of patients. The infection was controlled in 80% (21/26) of patients. Four out the five patients who had recurrent infection were type B hosts (p=0.63). The limb salvage rate was 89% (25/28). The overall complication rate was 32%.

**Conclusions:** The use of ACC-IMNs was an effective treatment strategy and associated with high limb salvage and bone union rates. Furthermore, the infection recurrence rate was low. Knee fusion patients after failed TKA should be counseled preoperatively for a potential high complication rate.

## Introduction

The management of osteomyelitis, a serious complication of orthopedic surgery, is often exacerbated by the presence of internal implants at the infected site. To control infection, the contaminated hardware is removed, and external fixation devices have been used to stabilize the long bone segments 4. The rationale behind this strategy, as opposed to repeat internal fixation, is to prevent bacterial colonization around new internal implants which could otherwise continue to hinder bony union. However, external fixation has its own drawbacks including patient discomfort over prolonged treatment courses, difficulty walking and maneuvering, and pin site infections 9. In an effort to avoid external fixation, staged sterilization of the intramedullary (IM) canal was introduced using an antibiotic loaded, cement coated wire to control infection followed by definitive, locked IM nailing. Wasko et al 28 treated 10 patients with fracture related infection (FRI) using antibiotic cement rods that were fabricated over a k-wire. Patients underwent a second procedure to remove the cement implant and provide definitive fixation 6 weeks after the initial surgery. The authors reported midterm results of 100% infection control and bone union. This method requires two surgeries and carries a risk of later infection from the uncoated implant used at the second stage 12,28.

Over the past decade, the use of internal fixation with antibiotic cement coated locked intramedullary nails (ACC-IMNs) has been proposed 1,3. The advantages of this technique include infection control with immediate stability from the locked IM nail, a single stage surgical intervention, and avoidance of an external fixator. Many authors have reported on the effectiveness of this technique 3,6,8,10,18,21,22,26,28. However, there exists variation in the techniques described and in the results obtained among these reports with regard to union, revision, and recurrence rate. Additionally, most reports shared one common limitation, which was the small sample size of patients included in the study and brief follow up periods. The goal of our study is to report on relatively larger consecutive series of antibiotic cement-coated interlocking intramedullary nails for treating septic complex lower extremity reconstruction with a minimum of 2 year follow up.

## Methods

After obtaining the institutional review board approval, we retrospectively reviewed the medical and radiographic records for all consecutive patients from January 2010 to August 2017 who underwent a limb salvage procedure with ACC-IMNs. Patients with follow up periods of less than 2 years were excluded from the study.

**Figure 1:**
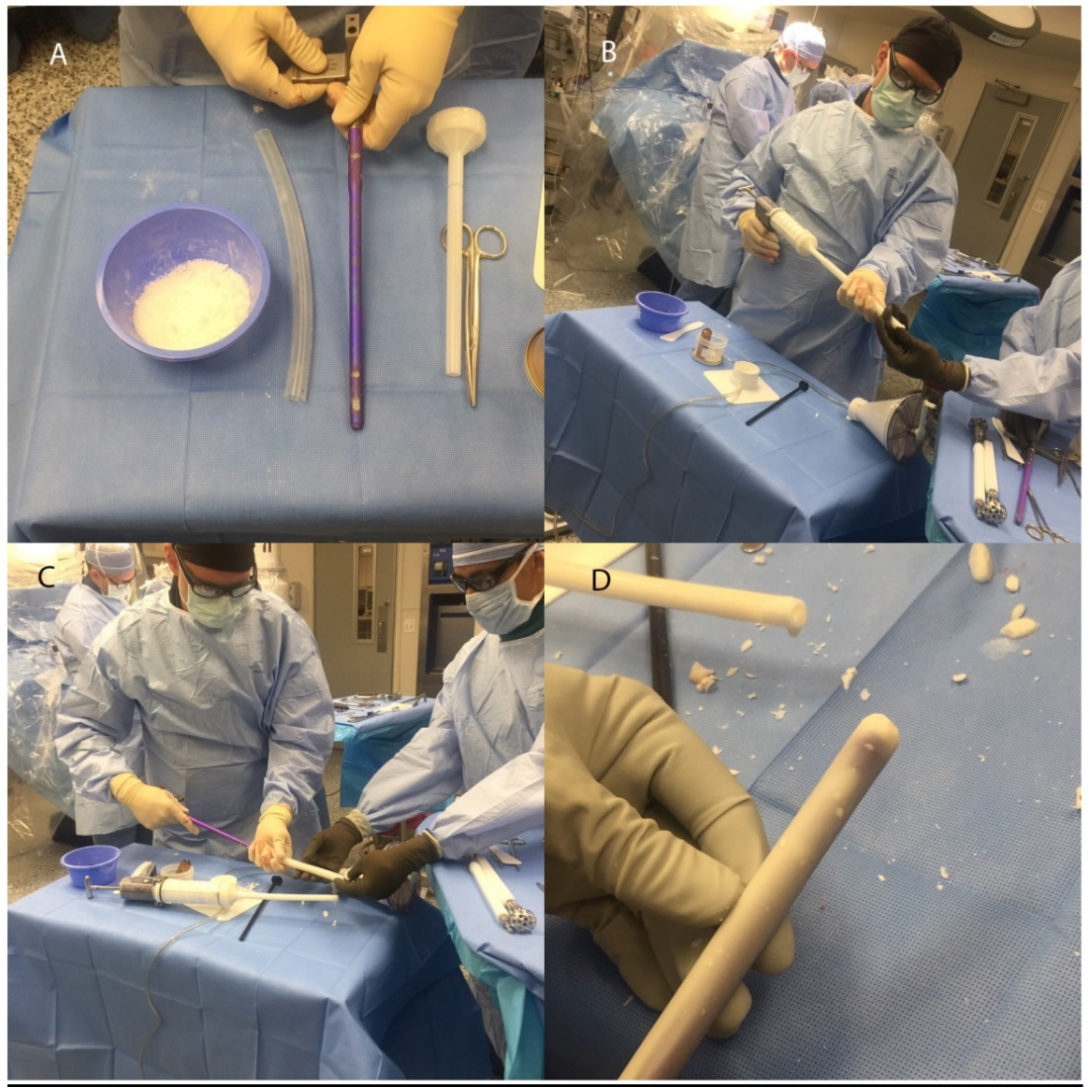
Photographs demonstrating the technique of coating intramedullary nails with antibiotic cement. **(A)** The nail is assembled, bone wax is placed into the holes, and the silicon tubing is cut to length. **(B)** The antibiotic is mixed into the cement (often requiring more liquid monomer), and mixture is pumped into a silicone tube using a cement gun. **(C)** The nail is then inserted into the silicone tube while the cement is still wet. An assistant plugs the distal end of the tube to avoid excessive cement leakage and maximize the cement volume around the nail. **(D)** Once the cement is hardened, the mold is then removed, and the nail tip is fashioned into a bullet shape making it ready to insert. We typically over ream the canal by 2 mm more than the outer cement coating diameter. This would prevent delamination of the cement mantle when the nail is pushed into the canal.

We reviewed patients' demographics, indications, and the involved anatomical sites. In addition, we also reviewed the preoperative infecting organism (based on culture results), host type (based on Cierny-Mader classification) 2, time to bony union, need for revision procedures, limb salvage rates, and recurrence rates were recorded. In all patients, infection was either suspected or confirmed pre operatively. The criteria for infection included the presence of drainage, cellulites, elevated inflammatory markers [erythrocyte sedimentation rate (ESR) and C-reactive protein** (**CRP)] and radiographic findings of osteomyelitis. This was further confirmed with five intraoperative tissue culture samples were sent to guide postoperative systemic antibiotics. Wound debridement was then thoroughly performed down to visibly bleeding bone. Standard locking intramedullary nails were used for all patients, and the antibiotic coating (consisting of antibiotic loaded polymethyl methacrylate (PMMA) from Simplex (Stryker, Kalamazoo, MI)) was customized and applied to the implant in the operating room. Ideally, the antibiotics would be tailored to previous culture results. In this series, the antibiotic cement formula we used consisted of mixing 2g of vancomycin and 2.4g of tobramycin per bag (40g) of Simplex cement with Tobramycin (1g) (for a total dose of 3.4g Tobramycin). We used commercially available silicon tubing with an inner diameter of 12.7 mm and used 10 mm diameter IM nails to fabricate the nail as described previously 14 (**Figure 1**).

In all cases, an infectious disease specialist prescribed culture-specific intravenous (IV) antibiotics for the duration of 6 weeks followed by culture specific oral antibiotics until bony union or joint fusion which has been the protocol at our institute. Patients were monitored clinically and via laboratory testing every week after surgery during systemic antibiotic administration. The infection was considered controlled by the absence of clinical infection (drainage, cellulitis and warmth) without any antibiotic treatment for at least two weeks. Patients were followed far beyond two weeks with a minimum of two years. Laboratory values were not followed beyond the antibiotic treatment course regularly. If the infection was thought to be poorly controlled or recurring then serum ESR and CRP were obtained. Patients were considered to have bone union/fusion once three of four cortices were united on radiographs and the patient was pain free when bearing full weight. Clinical follow up then was every 3 months in the 1^st^ year and every 6 months during the 2^nd^ year after surgery.

Functional outcomes were assessed using the Association for the Study and Application of Methods of Ilizarov (ASAMI) criteria with the modification of adding amputation to the score as a “failure” (20) (**Table 1**). Fusion surgery patients could not qualify for an excellent score due to joint motion restriction.

### Statistical analysis

Descriptive statistics in the form of means and ranges were utilized. The two-tailed Fisher exact test was used to compare categorical variables. The P value < 0.05 was considered statistically significant. The statistical package for the social sciences (Inc., Chicago, IL, USA) version 20.0 was utilized for the statistical work.

## Results

**Figure 2:**
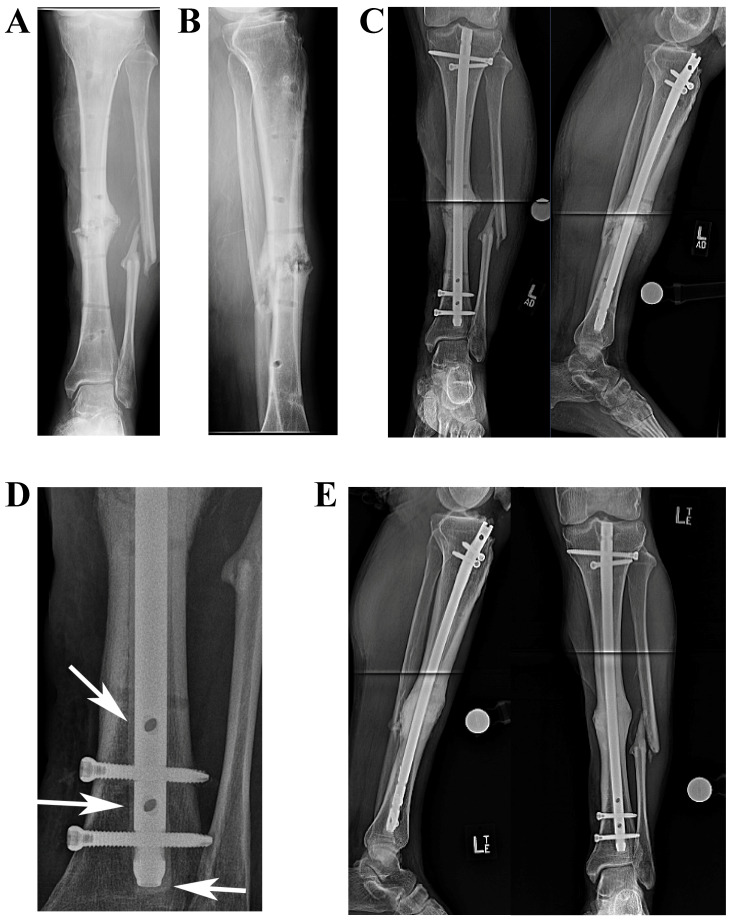
Serial radiographs showing a septic tibial nonunion patient who failed debridement, antibiotics, and external fixation treatment. **(A)** Preoperative anteroposterior (AP) view illustrating a midshaft tibial nonunion **(B)** Preoperative Lateral view of the tibia illustrating a midshaft tibial nonunion. He was treated with repeat open debridement, culture specific antibiotics, and ACC-IMN. **(C)** Early postoperative AP and lateral radiograph showing the ACC-IMN. **(D)** This close up view illustrates the antibiotic cement coating around the IMN (arrows). **(E)** AP view of the tibia showing full bony union with the ACC-IMN in place (left image). Lateral view of the tibia showing full bony union with the ACC-IMN in place (Right image).

A total of 28 patients were eligible and included in the study. There were 16 males and 12 females with a mean age of 62 years (range 22-88). The mean follow up period was 40 months (range 28-84). Using ACC-IMNs, 14 patients (50 %) underwent knee fusion after infected total knee arthroplasty (TKA). All of these patients had failed at least one two-stage revision knee arthroplasty surgery to control the infection and were indicated for knee arthrodesis. Of these 14 patients, 4 patients had an artificial knee fusion with antibiotic cement spacer used to bridge large bone defects (14). The ACC-IMN was used in 8 patients (28%) secondary to septic tibial nonunion (**Figure 2**). Seven of these patients were diagnosed with FRI and one patient had an oncologic infection with a history of squamous cell carcinoma excision and radiation therapy that progressed to tibial osteomyelitis. The ACC-IMN was used in 6 patients (21%) with ankle fusion nonunions. All of the ankle arthrodesis patients shared a post traumatic etiology requiring fusion: Two of these patients had infected Charcot ankles, 3 patients had infected pilon nonunions requiring arthrodesis reconstruction (two were treated for suspected FRI and the other for confirmed FRI), and one patient had a previously infected triple arthrodesis performed for post traumatic arthritis.

Ninety two percent of patients (26/28) had a confirmed infection with positive intraoperative cultures with *Methicillin-Resistant Staphylococcus Aureus* (MRSA) being the most commonly cultured organism (10/26). The two patients with negative intraoperative cultures were treated identically with IV antibiotics for suspected infection (**Table 2**). Bony union/fusion was achieved in 87% (21/24) of patients with a mean time to consolidation of 20 weeks (range12-40) after surgery. Knee fusion was successful in 8/10 (80%) of cases, ankle fusion was successful in 6/6 cases, and tibia union was successful in 7/8 (87%) cases. The infection was controlled in 80% (21/26) of patients: 11/14 (79%) knee fusions, 6/6 ankle fusions and 6/8 (75%) tibia cases. Of the five patients where this technique was unable to control infection, one remains on chronic antibiotic suppression. Three were treated with above knee amputation and one with segmental resection and bone transport. All of these four patients have infection control off of antibiotics at latest follow up.

Sixty seven percent of patients (19/28) were type B hosts while the remaining 33% were type A (9/28) hosts. The rate of recurrence of infection was 20% (5/26). Four out of the five patients (80%) who had a recurrence were type B hosts, and one patient (20%) was a type A host. In our study, host type was not statistically significant (P=0.63) related to recurrence. Patients had a stable limb during the last follow up visit with a limb salvage rate of 89% (25/28). Three patients from the knee fusion cohort who underwent above knee amputation were type B hosts that had persistent uncontrolled infection. Two of them also had a persistent nonunion at the knee fusion site in addition to the infection.

Nine patients (32%) had postoperative complications that are reported in detail in **Table 3**. ASAMI scores indicate that most patients in the knee and ankle fusion groups had good outcomes (any fusion procedure precludes an excellent score due to joint motion loss), while patients in the tibial group had mostly excellent to good outcomes. Detailed ASAMI scores are reported in **Table 4**.

## Discussion

The role of ACC-IMN in treating difficult bone infections has emerged in multiple reports. One major advantage of this technique is providing bone stability while delivering local antibiotics in single stage intervention. In our report, infection was controlled in 80% of cases and union was achieved in 87% with 89 % limb salvage rate at the latest follow up with MRSA being the most cultured organism in our population. These results are in-line with success rates reported for the treatment of septic nonunions using the Ilizarov external fixator where patients can spend a mean of 344 days in a frame 23. In another study, union rates were 93% (15/16) with using bifocal compression-distraction for large infected defects but with the drawback of a prolonged time needed in the fixator 15.

There have been controversies in the literature on whether to use IMNs in the presence of virulent organisms or polymicrobial infections. Many authors suggested that the presence of internal implants in such circumstances poses a risk of development of deep infection 5,7,13,29. In contrary, our results highlight the feasibility of controlling infection in a single stage intervention despite the presence of virulent organisms with MRSA being the most cultured organism in our population. However, our results should be interpreted with caution as knee fusion patients had 50% (7/14) complications rates with 3 patients who underwent above knee amputation. In this population, four patients underwent artificial knee fusion. This technique includes bridging the bone defect with a cannulated (to allow for the ACC-IMN passage) antibiotic cement spacer. The artificial knee fusion technique has been used in elderly patients with massive bone defects (generally >6 cm) after multiple revisions for failed TKA 14. One of these 4 patients failed due to persistent infection and underwent above knee amputation. Ten patients underwent ACC-IMN with intended knee bony fusion. Two of these have failed due to recurrent infections and nonunion at the fusion site and underwent above knee amputations. Schwarzkopf et al 25 found similar complication rates in knee fusion patient population after failed TKA. The authors found a 46.5% (20/42) complication rate with 4 patients needing repeat knee fusion and 2 patients undergoing amputation. The overall fusion rate in their study was 75% (30/42). These results represent the challenge of treating this clinical problem and may help surgeons to counsel patients preoperatively in this population.

Conway et al 3 reported a large cohort of patients who underwent ACC-IMN for infected fusions (group A, n=67) and infected nonunions (group B, n=43) with a minimum follow up of 18 months after surgery. The most commonly cultured organism in both groups was MRSA. The authors achieved bone fusions with limb salvage in 93% of patients in group A and 100% in group B. Five patients of the infected arthrodesis group (group A) underwent amputation due to persistent infection and nonunion. Our findings are in-line with these results. A difference noted in our series when compared with Conway et al is our use of Simplex with Tobramycin bone cement instead of the Palacos (Heraeus Medical, Yardley, PA) used by the other authors. Palacos has been shown to exhibit a superior elusion profile but had the complication of routinely delaminating due to its brittle mechanical properties 11. This study shows that Simplex can provide similar microbial control. Although not formally studied, Simplex rarely delaminates upon nail insertion or removal. We believe that coating the IMN with antibiotic cement reduced the burden of multiple surgical procedures as well as the use of external fixation devices. However, we acknowledged that regardless of the technique used, the host susceptibility plays a major role in the success of treating the infection 12. In our study, a high percentage of the recurrent infection cases were type B hosts. Another limitation of ACC-IMN technique is that the antibiotic used in the cement is typically selected empirically. Antimicrobial choice is best made based on previous culture results when available and may be dictated by the need for heat stable molecules. Nevertheless, if we compare ACC-IMN technique with antibiotic cement alone to the local effects of spacers, the release of local antibiotics is effective in the first weeks. The ACC-IMN is intended to limit the risk of failure to treat the infection by avoiding early fixation of a residual post-surgical inoculum on the internal material. It therefore increases the chances of success of the "single stage". On the mechanical level, it optimizes the stability of the assembly (over a flexible nail) and increases the chances of consolidation. From these points of view (mechanics and the treatment of infection), this technique seems well suited for septic non-unions of long bones or for treatment at the ankle. In contrary, the stabilization of a knee arthrodesis remains a difficult problem. These are patients with limbs that will likely be amputated without successful knee arthrodesis. Multiple prosthetic revision surgeries have excavated all cancellous bone surfaces, leaving a thing shell of bone at the fusion site. Autologous bone grafting is impossible and allograft serves as an optimal nidus for infection. Treatment with external fixation must include transcutaneous Schantz pins through swollen thigh tissue with prolonged, painful courses wearing the frame. Union is often partial and often requires subsequent internal fixation. Any intramedullary technique is welcomed by patients and surgeons alike. Other internal stabilization techniques, such as modular knee arthrodesis nails with anti-microbial coating, can be promising and should be explored and studied. However, these implants must be maintained for life with associated concerns over periprosthetic fractures or eventual re-infection and are extremely difficult to remove when infected requiring complete take down of any fusion achieved. In our series, 79% of knee arthrodesis cases united and were infection free without the need for an external fixator which is a major accomplishment given the gravity of this problem.

Time to union was an average of 20 weeks (range 12-40). There were 6 patients that required greater than 20 weeks to unite: 5 were in the knee fusion cohort where judging union is particularly difficult and delayed healing is typical; one was in the tibia nonunion group where a failure to follow up for several months led to the discovery of union at 40 weeks.

The recent literature has shown promising results with the use of antibiotic cement coated rods (**Table 5**) 3,8,12,18,21,22,26. Lam et al 12 recently investigated the effectiveness of limb salvage reconstruction in 67 patients with chronic tibial and ankle osteomyelitis using many techniques, including ACC-IMN. The authors used the Fracture-Related Infection (FRI) classification 17 to label their patients as confirmed or suspected FRI. Eighty eight percent of patients had confirmed infection and 12% had suspected infection. Ten patients (15%) were treated with ACC-IMN. The overall infection control was 91% with 96% limb salvage rate. The authors found that patients with history of diabetic neuropathy and patients with a higher number of repeat salvage surgery attempts were more likely to fail. Metsmakers et al 18 used a gentamicin-coated intramedullary tibia nail (Expert Tibia Nail (ETN) PROtect™) for the surgical treatment of complex open tibia fracture and revision cases. The implant was coated with a layer of poly (d,l-Lactide) (PDLLA) impregnated with gentamicin. These nails were used in 5 tibial FRI patients and 11 patients with acute open fractures. All patients had no evidence of deep infection at the final follow up. However, 4 patients (25%) underwent further procedures due to nonunion. Pawar et al 21 studied the use of ACC-IMNs in seven infected Charcot ankles after failed circular fixator treatment in five of these. In all cases, the infection was eradicated and bony union was achieved after a mean time of 4 months. Taken together, the use of ACC-IMNs can provide satisfactory results when dealing with infected long bone or joint infections in the lower extremity. Most importantly, coating interlocking intramedullary nails can provide immediate stability while delivering local antibiotic treatment. In our series of 8 tibial septic nonunions treated with this method, 2 patients had recurrent infection. One was treated with a large resection of necrotic bone and a trifocal bone transport. This sequence suggests that the patient was a poor candidate for ACC-IMN and should have undergone bone transport. It also underlines the point that although local antimicrobial therapy can play a role in the management of infection, a thorough debridement of all dead and poorly vascularized tissue is a critical element for success 16. The second patient was implant free and appeared to be infection free for 2 years and then presented with skin breakdown and Cierny-Mader type 3 surface osteomyelitis penetrating the IM canal. Although the bone was united, he was a late failure of the ACC-IMN and was treated with local debridement, local antibiotic PMMA in the IM canal, and free flap coverage to replace the chronic poor skin over the medial tibial face. He has had no further infection on suppressive oral antibiotics over the past 6 months.

Our study has several limitations. Although our sample size is relatively large when compared with the most reported literature (excluding Conway et al study 3), the heterogeneity in our sample may be pose limitations for each specific anatomical entity. In addition to that, the study is limited due to its retrospective nature with the absence of a control group. Furthermore, despite using nonparametric statistical test (2-tailed Fisher Exact) to compare categorical variables, the number of patients with recurrent infection was low to show a difference and underpowered and does not allow for firm clinical conclusion. One challenging factor is that such patients' population is uncommon, and therefore, designing a prospective study with large number of patients may require multi-institutional efforts. It has been recently discussed in multiple international guidelines, practically all surgeons that treat musculoskeletal infections use antibiotic loaded bone cement (PMMA). Nevertheless, the use of PMMA has important downsides. van de Belt et al 27, for example, evaluated the release profiles of 6 types of bone cements in vitro and found that the released antibiotic fell below the detection limit after 1 week and only 4%-17% of the incorporated antibiotic was released. In a clinical study by Neut et al 19, the authors retrieved gentamicin-loaded PMMA beads after revision surgery for PJIs. Cultures were positive for bacteria on gentamicin loaded beads in 90% of the patients. A significant amount of these strains proved to be gentamicin resistant, which raises concerns over the development of antibiotic resistance due to prolonged release at sub therapeutic levels. Therefore, there is a clinical need for coating technologies that markedly improve the safety and efficacy of local delivery 16,24. Finally, the use of six weeks of IV antibiotic therapy is still customary in the United States but has been challenged and is no longer standard in most of the world. It is not the intension of these authors to recommend widespread adaptation of the systemic antibiotic protocol presented here.

In conclusion, the use of ACC-IMNs was an effective strategy and associated with high limb salvage rates, unions and infection control rates. Our results have also shown that this strategy is effective despite the presence of virulent organisms. Knee fusion patients after failed TKA should be counseled preoperatively for a potentially high complication rate.

### Level of evidence

Prognostic Level IV.

## Figures and Tables

**Table 1:** Functional ASAMI scoring

Functional classification	Results
Excellent	Ability to perform activity of daily living (ADL) with no pain and no limp, loss of ankle motion > 5 degrees and loss of knee motion less than 15 degrees, no complex regional pain syndrome (CRPS).
Good	Almost all ADLs with minimal pain and minimal limp, joint stiffness, no CRPS.
Fair	Most ADLs with minimal pain, a limp, joint stiffness, no CRPS
Poor	Significantly limited ADLs, pain requiring narcotics, CRPS.
Failure	Amputation
**Patients who underwent knee or ankle fusions will not qualify for excellent classification due to the loss of motion at the knee or ankle with intended fusion. These patients will be graded with a scale that ranges from good to failure.**	

**Table 2:** Results of intraoperative cultures

Cultured organisms	Number of Patients
Corynebacterium	1
E. Coli	1
Enterococcus	1
MRSA1	10
MRSE2	1
polymicrobial	6
Pseudomonas Aeruginosa	1
Staphylococcus Epidermidis	2
Staphylococcus Lugdunensis	1
VRE3	2
Total	26
Negative Cultures	2

^1^Methicillin-Resistant Staphylococcus Aureus; ^2^Methicillin-Resistant Staphylococcus Epidermidis; ‪^3^Vancomycin-Resistant Enterococcus.

**Table 3:** Reported complications in our study population

Complications	
Treatment Group	Description
Knee fusion	Delayed union that required bone marrow aspiration injection (N=1)
	Distal locking Screws Back out required operative intervention (N=2)
	Incision/Drainage to treat Purulent Drainage 12 days post-surgery (N=1). No infection recurrence at final follow up.
	Persistent infection in 3 patients with two of them having nonunion and progressed to Amputation (N=3).
Tibial Septic Nonunion	Persistent Septic Nonunion, Required Trifocal Bone Transport with a circular frame device (N=1)
	Recurrent infection with bony union (N=1) 2 years after surgery. Treated with debridements, local antibiotic, and free flap for improved skin coverage- no further infection
Ankle fusion	No reported complications.

**Table 4:** ASAMI Function Score by Treatment Group

Treatment group	Functional ASAMI Score				
	Excellent	Good	Fair	Poor	Failure
Knee fusion (N=14)	0	6	5	0	3
Tibial Septic Nonunion (N=8)	2	5	1	0	0
Ankle Fusion (N=6)	0	3	3	0	0

**Table 5:** Literature review

Study	Number of patients	Infection control	Union or fusion rate	Limb Salvage rate	Mean follow up period in months (range)
Makhdom et al 2020 (current study)	28	80%	87%	89%	40 (28 to 84)
Lam et al 2019 (12)	67 (tibial FRI*, infected ankles and hindfoot)	91%	88.9%	92%	46 (13 to 177)
Conway et al 2014 (3)	67 (Infected fusions)	73% (27% recurrent infection)	93%	93%	31 (12 to 122)
	43 (infected nonunions)	70% (30% recurrent infection)	100%	100%	39 (12 to115)
Kanakaris et al 2014 (8)	24 tibial and femur FRI	96%	96%	96%	21 (8 to36)
Metsemakers et al 2015 (18)	5 tibial FRI	100%	75%	100%	Only minimum 18 months of follow up reported
Pawar et al 2013 (21)	5 Charcot infected ankles	100%	100%	100%	18 (12 to 24)
Qiang et al 2006 (22)	13 tibial FRI; 6 Femur FRI	94%	94%	94%	16 (6 to 28)
Thonse et al 2007 (26)	long bone FRI (n=11) and infected fusions (n=9)	95%	85%	100%	16 (7 to 40)

*Fracture-Related Infection.
